# Rising global burden of breast cancer: the case of sub-Saharan Africa (with emphasis on Nigeria) and implications for regional development: a review

**DOI:** 10.1186/s12957-018-1345-2

**Published:** 2018-03-22

**Authors:** Samuel O. Azubuike, Colin Muirhead, Louise Hayes, Richard McNally

**Affiliations:** 1 Institute of Health and Society, Newcastle University, The Baddiley-Clark Building Richardson Road, Newcastle upon Tyne, NE2 4AX UK; 2 Department of Public and Environmental Health, National Open University of Nigeria, Plot 91, Cadastral Zone, Nnamdi Azikiwe Express Way, Jabi, Abuja, Nigeria

**Keywords:** Breast cancer, Incidence, Mortality, Africa, Nigeria

## Abstract

**Background:**

Despite mortality from breast cancer in Africa being higher than in high income countries, breast cancer has not been extensively studied in the region. The aim of this paper was to highlight the rising burden of breast cancer with an emphasis on sub-Saharan Africa as well as trends, characteristics, controversies and their implications for regional development.

**Methodology:**

A review of published studies and documents was conducted in Medline, Scopus, Pubmed and Google using combinations of key words-breast neoplasm, breast cancer, cancer, incidence, mortality, Africa, Nigeria. Graphical and frequency analyses were carried out on some of the incidence and mortality figures retrieved from published papers and the GLOBOCAN website**.**

**Findings:**

Globally, about 25% and 15% of all new cancer cases and cancer deaths respectively among females were due to breast cancer. Africa currently had the highest age-standardized breast cancer mortality rate globally, with the highest incidence rates being recorded within the sub-Saharan African sub-region. Incidence trends such as inherently aggressive tumour and younger age profile had been subject to controversies. Certain factors such as westernized diet, urbanization and possibly increasing awareness had been implicated, though their specific contributions were yet to be fully established.

**Conclusion:**

Unless urgent action is taken, breast cancer will compound sub-Saharan Africa’s disease burden, increase poverty and gender inequality as well as reverse the current global gains against maternal and neonatal mortality.

## Background

Cancer can occur in different anatomical sites in the body. Its incidence and mortality differ across regions and between sexes. Globally, lung cancer has been the leading cause of cancer deaths among males. However, breast cancer remained the most frequently diagnosed cancer in women and the leading cause of cancer death worldwide, with an estimated 1.7 million new cases and 521,900 deaths in 2012 compared to 1.38 million new cases and 458,000 deaths in 2008 [1, 2]. This amounts to 25% of all new cancer cases and 15% of all cancer deaths among females [1, 3–5]. An estimated 231,840 (29%) new cases of invasive breast cancer were expected to be diagnosed among women in the US during 2015, compared with 105,590(13%) cases of lung cancer in the same population [6].

The World Health Organization had estimated that female breast cancer resulted in a total of 5,884,000 years of life lost globally during 2004 [7]. This represented just over 1% of all premature deaths amongst females, ranging from around 8% in parts of Europe to less than 0.5% in Africa [7]. Breast cancer ranked as the fifth cause of death resulting from cancer overall (522,000) [8]. Global trends suggested increasing incidence in the past three decades up to 2010, although some developed countries recorded trends lower than the global average [9]. However, over the past two decades, incidence and mortality trends had remained relatively stable and even decreasing in many developed countries. On the contrary, it had been on the rapid increase in many parts of Africa, Asia, central and South America [7, 10, 11]. The worrisome aspect however was the fact that while incidence in the African region (although rising) was lower than in other continents except Asia, its age-standardized mortality rate ranked highest globally with Nigeria, the most populous African nation, having the highest mortality rate. It has therefore become necessary that the attention of global and regional policy-makers, researchers and the general public be drawn to the growing danger that breast cancer could pose to Africa's development, especially in the sub-Saharan regions where little attention is being paid on non-communicable diseases, probably due to the high burden of communicable diseases. It was against this backdrop that this review was conceived to give an overview of the global burden of breast cancer with an emphasis on Africa, its sub-Saharan region and Nigeria. The aim was to highlight the trends, characteristics, controversies and determinants of rising breast cancer burden in the sub-Saharan African region and to discuss briefly the implications of this rising burden to regional development. It must be mentioned that sub-Saharan Africa is an extraordinarily large area in Africa that is economically diverse (see Fig. 7).

### Research question

Principally this review aimed to address the following questions:

1. Is the breast cancer burden in sub-Saharan Africa on the increase?
2. Are the characteristics and determinants of this burden clearly ascertained?
3. Will the rising burden of breast cancer pose any threat to sub-Saharan Africa's regional development?

These were judged by International Agency for Research on Cancer [1] ascertaining whether the available incidence and mortality figures were increasing over time; (ii) reviewing the level of consensus on the determinants of Africa's breast incidence among experts; and (iii) ascertaining whether rising breast cancer burden will really have any social or economic impact on the region, and to what extent.

## Materials and methods

### Search strategy

A literature review of published studies and documents in Medline, Scopus, PubMed and Google was conducted. The combinations of keywords-“breast neoplasm,+developed countries+developing countries, +female incidence as well as phrases such as ‘global breast cancer incidence’, Burden of breast cancer in Africa, in sub-Saharan Africa, and in Nigeria”, were used. Google scholar was used only to search for specific papers identified from the reference lists of published papers.

### Eligibility criteria

Studies included in the review were those published between January 2000 and May 2016. All publications before this period were excluded. Studies were included if they were either peer reviewed or published by Internationally acknowledged organization such as the World Health Organization or on websites run by credible organizations. Such studies also needed to have a relevant title, abstract and full-text contents.

### Study selection

**Fig. 1:**
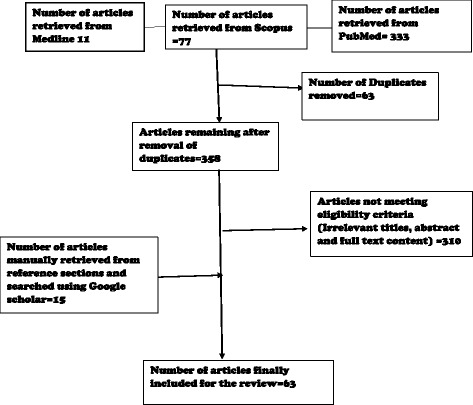
Diagrammatic explanation of data extraction process

The studies were selected based on the relevance of their titles, abstract and full-text contents, as summarised in the chart below (see Fig. 1):

### Data extraction

Data were retrieved based on their relevance in explaining global and regional incidence and mortality patterns. These were done principally from GLOBOCAN 2012 website and previously published papers. Maps showing breast cancer incidence and mortality in Africa, charts on United Nations world areas in relation to breast cancer incidence and mortality as well as chart showing the incidence and mortality among the top 20 countries of Africa were based on figures and analytical tools provided on the GLOBOCAN websites. The rest of the charts and tables were produced based on figures retrieved from previously published papers.

### Quality assessment

Studies included in this review were only those that met the set eligibility criteria which were determined prior to the commencement of this study. The quality, relevance and application of these studies were assessed by three independent experts in the field.

### Synthesis of results

Data inform of figures on the GLOBOCAN 2012 website and other previously published papers on breast cancer burden globally, in Africa and Nigeria were pooled to develop charts, graphs and tables that guided discussion. Furthermore, the results, opinions, and conclusions of previous studies were compared, contrasted and integrated to arrive at opinions, recommendation and conclusions in relation to the objectives of this review.

## Results summary

Globally, about 25 and 15% of all new cancer cases and cancer deaths respectively among females were due to breast cancer. Continents in the western world had higher age-standardized incidence rate of breast cancer compared to Africa and Asia. However, mortality statistics showed that Africa had the highest age-standardized mortality rate associated with breast cancer in the world. The result also provided evidence that the comparatively low incidence rate of breast cancer in Africa had been rising over the years. Though southern African sub-region had the highest incidence rate, western Africa could be said to have the highest burden when both incidence and mortality were considered. However, the highest increase in both incidence and mortality since 2008 were recorded in eastern and middle African regions. Controversies over the nature of breast cancer characteristic trend and the exact determinants were also suggested. There were evidence that the financial and other possible socioeconomic costs could be very high.

## Discussion

The results as subsequently discussed showed that even though the current assumption that Africa has comparatively low incidence rate of breast cancer could be said to be correct based on available statistics, this incidence rate is currently on the rise. What is missing most times in this narrative is the consideration of the possible impact of this seemingly low incidence rate on the National economies of African sub-regions if left unchecked. This is even felt more at the local communities and families who are often not protected socioeconomically by government policies. Breast cancer is like a death sentence in many African communities and families as shown by the high mortality rate—the highest in the world. These communities and families most times mourn and bear this burden alone as many of these cases do not come to the attention of national and international policy-makers. It was this need to draw attention to this rising burden in relation to its socioeconomic impact on national development that made this review necessary.

### Overview of global burden of breast cancer and trends

According to a systematic analysis of breast cancer in 187 countries, global breast cancer incidence increased from 641,000 (95% confidence interval (CI) 610,000–750,000) cases in 1980 to 1,643,000 (1421000–1,782,000) cases in 2010, an annual rate of increase of 3.1% [9]. It was the most frequent cause of cancer death in women in less developed regions (324,000 deaths, 14.3% of total), and—after lung cancer—is currently the second most frequent cause of cancer death in women in more developed regions (198,000 deaths, 15.4%) [8].

Incidence rates varied across countries with developed countries of northern America, Australia and New Zealand having a higher incidence compared to Africa and Asia [2].

**Fig. 2:**
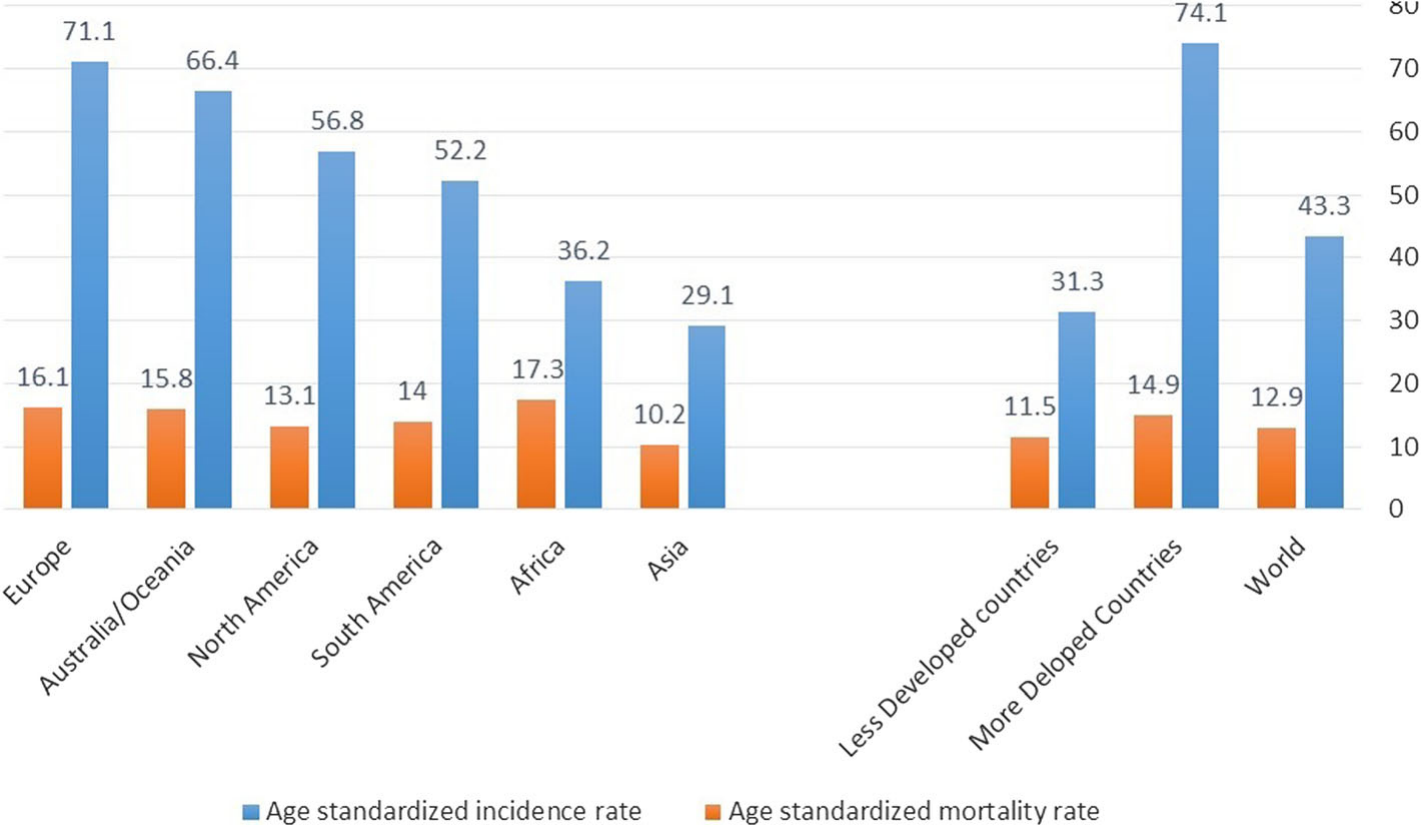
Global burden of breast cancer by continents (per 100,000 women per year)(based on Ferlay et al. [8]). Figure 2 was based on seven continent model [65]. Central America and Caribbean were grouped under northern America, while New Zealand, Polynesia and Micronesia were grouped under Australia. It showed that while continent of Europe had the highest ASR incidence rate, Africa had the highest ASR mortality rate. This may differ slightly when analysis is based on United Nation’s world areas shown

**Fig. 3:**
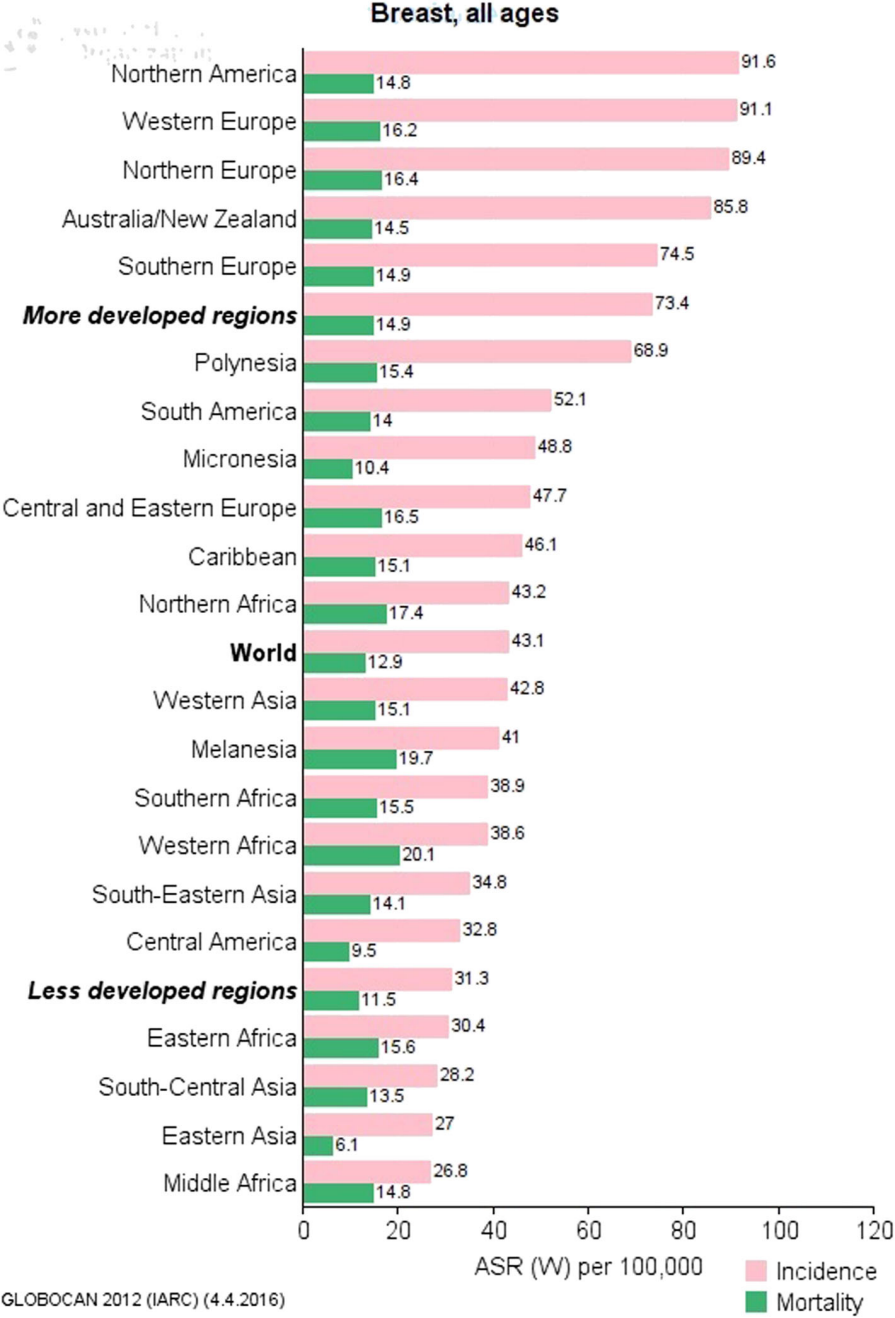
Breast Cancer Burden by United Nations world areas. The figure shows that while northern America has the highest incidence rate, western Africa has the highest mortality rate

Europe was the continent with the highest breast cancer incidence (Fig. 2). In terms of individual countries, Belgium and Denmark (119.9 and 105.0 per 100,000 respectively) topped the incidence table [4] while regionally the highest incidence of breast cancer was in northern America and western Europe. The lowest incidence was in eastern Asia and Central America (Fig. 3).

Although incidence rates remained highest in more developed regions, mortality was relatively much higher in less developed regions [4]. For example, in northern America, breast cancer incidence had reached more than 91 new cases per 100,000 women annually, compared with 27 per 100,000 in middle Africa. In contrast, breast cancer mortality rates in these two regions were almost identical, at about 15 per 100,000 [12]. While more developed countries account for about one-half of all breast cancer cases, they record only 38% of deaths [1, 5].

Literature suggested that trends in incidence varied with age and ethnicity in different populations. For example, it had been reported that women of reproductive age were twice as likely to develop breast cancer in developing countries as in developed countries, while more than two-thirds of cases in developed countries occurred in women of age 50 years and above [9]. In the USA, the breast cancer incidence rate decreased almost 7% among white women from 2002 to 2003 and remained stable from 2007 to 2011. However, it increased by an annual rate of 0.3% among black women between 2007 and 2011 [6]. Ethnic differences in mortality rates had also been reported in the USA, probably related to poor insurance coverage among ethnic minorities [13]. In addition, differences in age at breast cancer diagnosis by ethnicity had been reported in a number of countries, including Sweden [14] and Iraq [15] with immigrants and Kurds exhibiting earlier mean age at diagnosis than their matched Swedish and USA controls respectively.

### Breast cancer incidence and trends in sub-Saharan Africa

It was difficult to estimate exactly the incidence of cancer including that of breast in Africa. In the former volume (volume ix) of WHO publication on global incidence of breast cancer, data from only 5 out of the 14 African countries that submitted data were found acceptable, due to poor quality and incompleteness. This represented 31% (5/16) of cancer registries and covered only 1% (8.8 million) of the population [16]. The recent publication (volume x) recorded only an additional three countries [17].

**Fig. 4:**
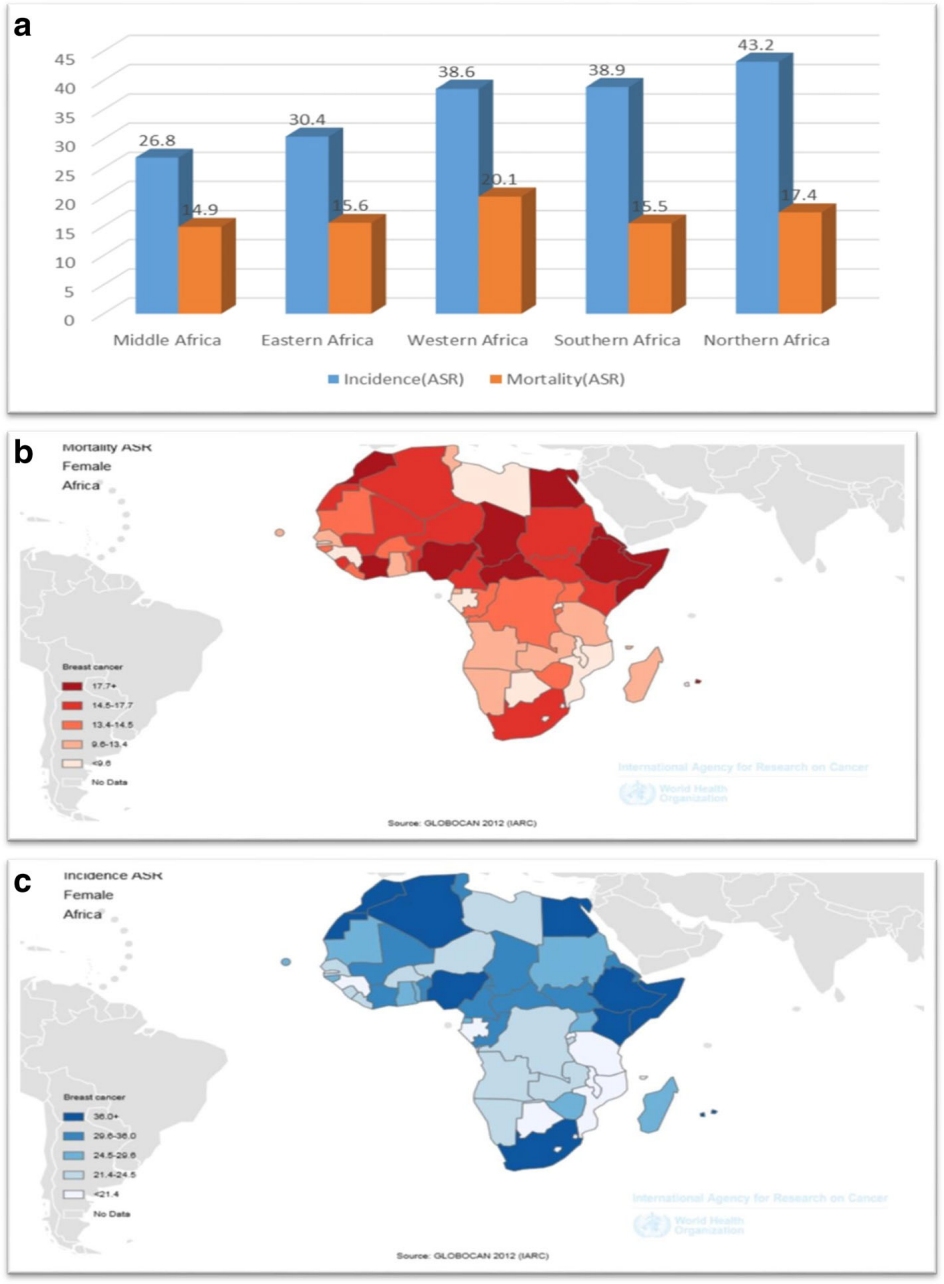
**a** Distribution of breast cancer incidence and mortality in Africa (based on GLOBOCAN, 2012). **b** Map of Africa showing distribution of age-standardized incidence rates by countries. **c** Map of Africa showing distribution of age-standardized mortality rates by countries

However, recent incidence data from registries in Uganda, Zimbabwe, the Gambia, and Mali between 1991 and 2010 [18–21] as well as GLOBOCAN figures (see Fig. 4a–c) provided strong evidence to suggest that breast cancer incidence in sub-Saharan Africa was on the increase. In Zimbabwe, for example, a 4.5% annual increase in incidence over the period 1991–2010 had been reported [20]. It should be reminded that these were only recoded incidents.

There was substantial variation in estimated breast cancer incidence across different African regions in 2012. Estimates of age-standardized incidence rates (per 100,000 women per year) as shown in Fig. 2a were 30.4 in eastern Africa, 26.8 in middle Africa, 38.6 in western Africa, and 38.9 in southern Africa [1, 18]. Hence, the rate was lowest in Middle Africa (26.8) and highest in southern Africa (38.9), probably due to high population of whites with higher exposure to economic and reproductive risk factors [22, 23]. With an average incidence of 33.7 per 100,000 women per year, sub-Saharan Africa tended to have a lower incidence rate compared to North Africa (43.2 per 100,000 women per year) [12]. However, country wise, the sub-Saharan African countries—Mauritius (64.2 per 100,000 women) and Nigeria (50.5 per 100,000 women) in eastern and western Africa respectively were ranked first and second in incidence ahead of Egypt (49.50 per 100,000 women) the highest ranked country of northern Africa, which occupied the third position [12]. In sub-Saharan Africa, breast cancer incidence (33.8 per 100,000 women per year) currently ranks only second to cervical cancer incidence (34.8 per 100,000 women per year), with only a small difference between these rates. Yet, this was not applicable to all the four regions of sub-Saharan Africa. Breast cancer incidence in western Africa in 2012 was 20.1 per 100,000 women per year compared to 18.5 per 100,000 women per year for cervical cancer. The gap was even more in southern Africa with 38.9 breast cancer cases per 100,000 women per year. The incidence rate for cervical cancer in southern Africa that same year was 31.5 cervical cancer cases per 100,000 women per year [12].

**Fig. 5:**
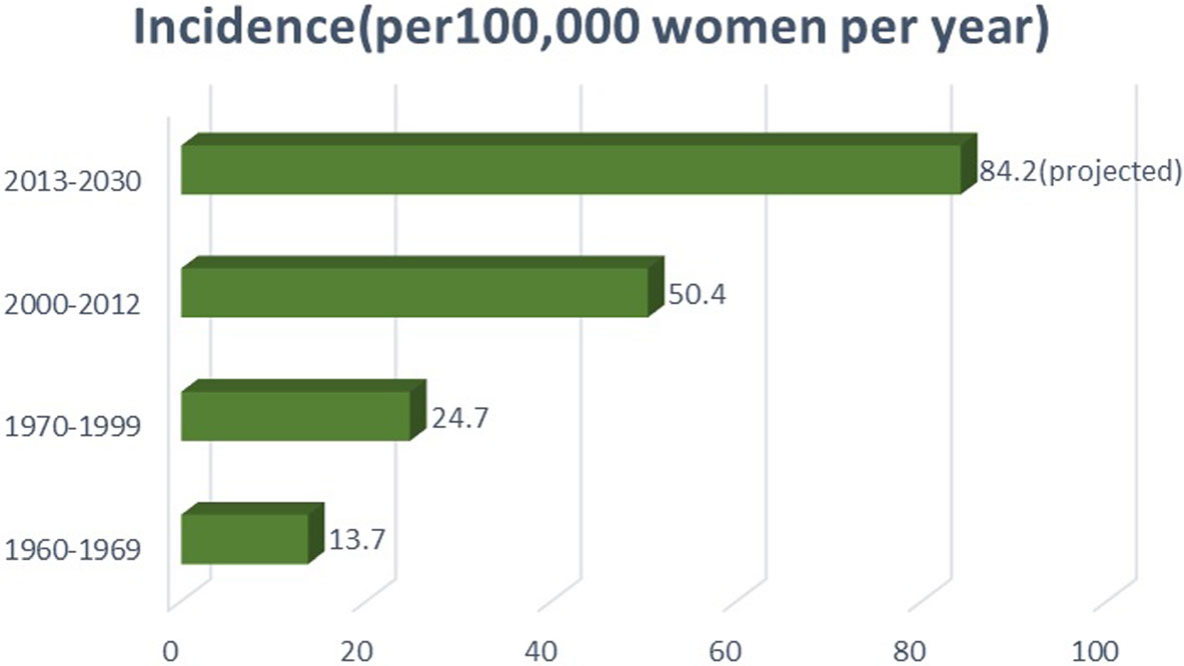
Increasing incidence of breast cancer in Nigeria since 1960 (based on Jeddy Egba etal [38] and GLOBOCAN, 2012 [12]

**Fig. 6:**
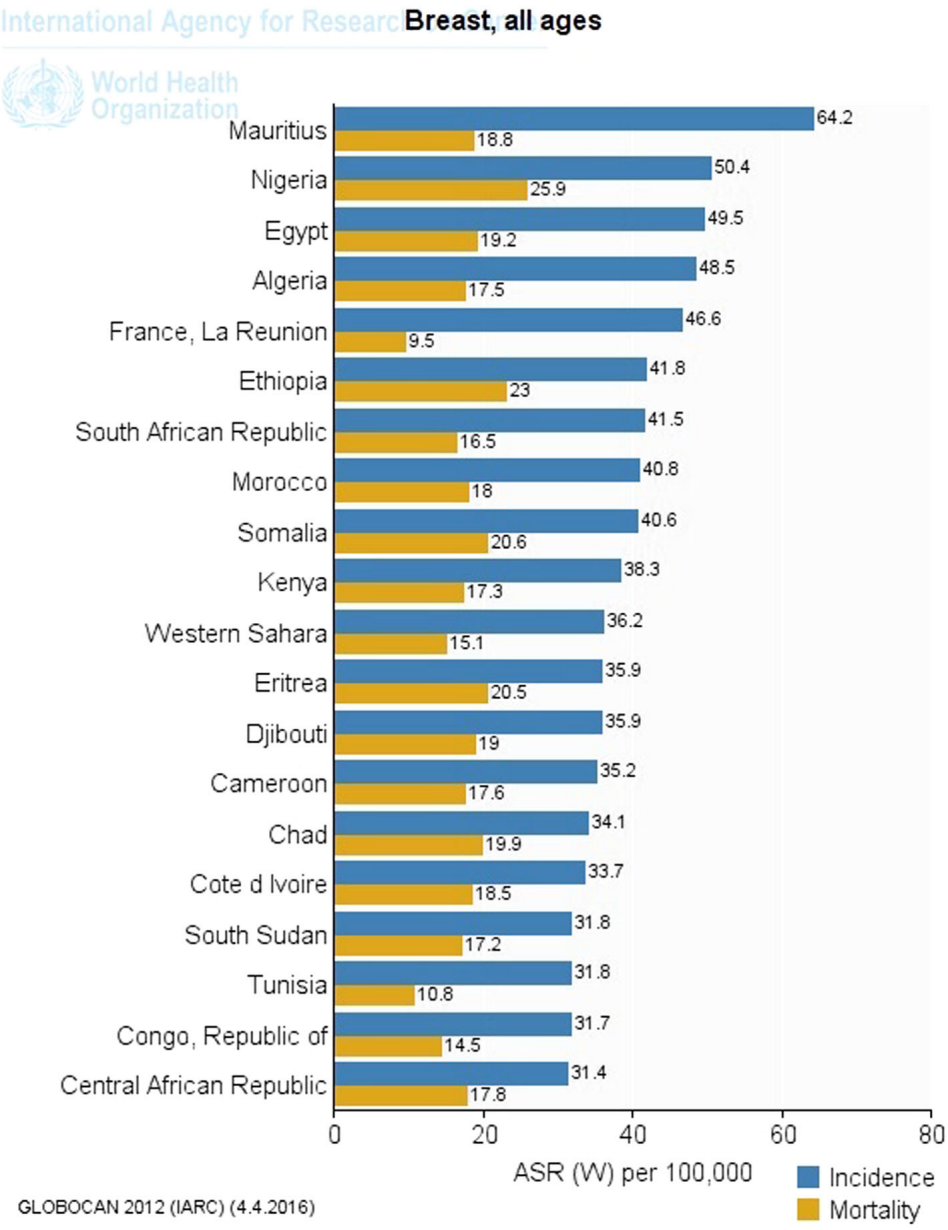
Top 20 countries with the highest incidence and Mortality associated with breast cancer in Africa

More disturbing was the suggestion that in contrast to the comparatively low incidence rate in Africa, the mortality rate might be disproportionately 50% higher relative to incidence rate [24, 25]. In comparison to other continents of the world, the age-standardized incidence rate of breast cancer for the African continent had been estimated to be the lowest (36.2 per 100,000 women per year) except for Asia (29.1 per 100,000 women per year). Yet, its age-standardized mortality rate (17.3 per 100,000 women per year) ranked higher than every other continent or region of the world. Though the region was projected to contribute 6.79% of the global incidence projection by 2030, it was estimated it would contribute a disproportionate 10% of the total mortality projected [12]. Sub-Saharan African data according to GLOBOCAN 2012 [12] showed that the highest age-standardized mortality rate was in western Africa (20.1 per 100,000 per year). This might be related to the high mortality rate in Nigeria. Middle Africa maintained the lowest position (14.9 per 100,000 per year) while eastern Africa had a rate of 15.6 per 100,000 per year. In consistent with the incidence trend, although cervical cancer remained the leading cause of cancer death among women in sub-Saharan Africa, breast cancer has remained the leading cause of death among women in the West African sub-region with an age-standardized mortality rate of 20.1 per 100,000 women per year in 2012 compared to 18.5 per 100,000 women per year for cervical cancer [14]. Breast cancer might constitute a higher threat to improved access to health care especially among women in sub-Saharan Africa if no action is taken.

It is important to note that even the comparatively lower incidence and mortality rates observed in eastern and Middle Africa might not imply a lower risk in its true sense. Table 1 suggested that the percentage increases in the annual standardized rate (ASR) for incidence and mortality between 2008 and 2012 were higher in those regions compared to southern and western Africa. This gives a cause for concern in the light of the fact these regions still had the highest incidence and mortality recorded for another important female cancer- cervical cancer. It was not quite clear whether this could be attributed to improved detection or increasing exposure to risk. Southern Africa was the only region with a comparatively stable incidence rate and interestingly a remarkable reduction in age-standardized mortality rate (Table 1). This might be associated probably with an influence from improved health care systems and better insurance coverage in South Africa. The reduction in mortality rate in South Africa as observed by Cubash et al. [26] could also reflect improvement in life expectancy resulting from recorded improvement in HIV/AIDs treatment especially among breast cancer patients infected by the virus.

**Table 1:** Estimated changes in ASR between 2008 and 2012 (computed based on Ferlay et al. 2008 [2] and 2012 [12] figures)

Sub-regions	Incidence			Mortality		
	2008	2012	%increase	2008	2012	% increase
Eastern Africa	19.3	30.4	36.5	11.4	15.6	26.9
Middle Africa	21.3	26.8	20.5	13.1	14.9	12.1
Northern Africa	32.7	43.2	24.3	17.8	17.4	− 2.3
Southern Africa	38.1	38.9	2.0	19.3	15.5	− 24.5
Western Africa	31.8	38.6	17.6	19.0	20.1	5.5

The table suggested that while higher age-standardized incidence and mortality rates remained higher in southern and western African sub-regions in both years, higher increases in both incidence and mortality rates were observed in eastern (36.5 and 26.9%, respectively) and middle (20.5 and 12.1%, respectively) Africa in 2012 compared to 2008. Southern Africa had a comparatively more stable incidence rate (only 2% increase) and interestingly a remarkable decrease in mortality rate

### Incidence trend characteristics and controversies

Existing evidence suggested that breast cancer in Africa tended to occur in relatively younger age groups and among premenopausal women compared to the western population [21, 27–31]. For example, a low mean age of 48 years among Nigerians and a median age of 46 years among blacks compared to 67 years among white British women had been reported [28, 32]. However, recent reports seemed to suggest a changing trend with higher incidence being reported among older and postmenopausal women [18–20], probably due to a changing demographic trend. Late presentation and advance stage distribution was common [27, 33, 34]. There had been suggestions of the existence of a possible inherently aggressive tumours biology similar to those observed among African American women [33, 35]. However, in the view of Akarolo-Anthony et al. [36], the dominant young age profile of cases, the predominant aggressive clinical course, and the high fatality rate that characterized African breast cancer cases might reflect the demographic structure of the African population (which tends to be younger) rather than intrinsic biological vulnerability as had been suggested [36]. They also queried the suggestion that African breast tumours were predominantly receptor poor, attributing it to possible problems arising from poor preparation and fixation of tissue samples, as well as lack of methodological standardization issues observed earlier by Adebamawo et al. [37]. However, the results of breast cancer trend analyses between 1987 and 2009 in Mali and Gambia by Sighko et al. [21] could not attribute the increase in incidence of pre-menopausal breast cancer recorded in that study to changes in life expectancy and population structure. They argued that while a small but significant shift in incidence towards older age for other cancers such as stomach cancer in women of the same population was observed, occurrence of breast cancer was dominantly among younger people (< 50–55 years). It seemed therefore that the picture of breast cancer incidence in Africa is still unfolding, and it might take some time before a valid conclusion could be drawn. There is a need for the promotion of more quality studies in order to determine exactly the true picture of breast cancer incidence in sub-Saharan Africa and by extension the entire continent.

### Determinants of the rising incidence of breast cancer in Africa

One key factor that had been implicated in the increasing incidence of breast cancer in Africa and Nigeria in particular was the increasing adoption of westernized lifestyle and behaviours resulting in changes in diet, later age of reproduction and reduction in physical activity among the African population [2, 19, 20, 33]. This seemed to have been associated with rural-urban drift and socioeconomic improvement, as well as other urban-related changes that might have altered pre-existing protective characteristics of such cities; such as consumption of traditional and unprocessed foods, increased physical activity, and low exposure to carcinogens [38].

Some experts had attributed the rising incidence to increasing population and life expectancy with many African women living long enough for the disease to manifest [36, 39]. It had also been suggested that it might reflect a growing awareness of breast cancer and change in health seeking behaviours, as well as improvement in screening and diagnostics technology. However, many studies had reported poor awareness and poor availability of screening services, diagnostic skills and technology [7, 40]. While it may be obvious that the answer lies in a combination of these factors acting at various levels of the society, determining the differential contribution of each factor is necessary for the purpose of prioritizing intervention in the face of scarce resources. It might be against this backdrop that Sigko et al. had suggested that due to the conflicting results and controversies, further studies were needed in the region to determine whether this increasing trend could be associated with changes in reproductive, nutritional and environmental risk factors or sociological changes that might have increased awareness, recognition and diagnosis of the disease [21]. This suggestion is unarguably very timely and demands a sense of obligation on the part of International and national policy-makers as well as researchers in the region.

## Nigeria scene

Advanced stage distribution characterised the presentation of breast cancer in Nigeria like other parts of Africa, with more than 70% presenting at stage III or IV in some cases [27, 29, 33, 41] compared to ≤ 46% in Europe [32, 42]. This had been attributed to late presentation and delay in seeking treatment [33].

The population of Nigeria not only plays a major role in the incidence records of sub-Saharan Africa, but also provides an opportunity for reduction of breast cancer incidence and mortality in the region. Nigeria is the most populous country in Africa, with approximately 20% of the continent’s population and slightly more than half that of West Africa [39, 43]. It was therefore not surprising that of the estimated 681,000 new cases of cancer that occurred in Africa in 2008, Nigeria contributed 15% [43], with about 100,000 new cases of cancer occurring every year [2]. This figure likely underrepresented the true number of incident cases of breast cancer since they were mostly based on reports from medical institutions as opposed to incidence studies; nonetheless, this suggested that a substantial reduction in the breast cancer burden for all of sub-Saharan Africa could be achieved if breast cancer burden in Nigeria is reduced.

Nigeria, like other African countries, suffers from the problem of data unavailability. For example, of the eight volumes of estimates produced by WHO on cancer incidence globally, Nigeria only featured in the first three, with the last being volume III (1960–1969) [16]. However, Okobia et al. [44] reported that incidence doubled from 15.3 per 100,000 in 1976 to 33.6 per 100,000 in 1993 with a proviso that this might be an underestimate. A recent analysis of two population-based breast cancer registries covering 2.5% of Nigerian population, aimed at determining the cumulative incidence rate from 2009 to 2010, had been reported [39]. The combined ASR of breast cancer incidence from both registries was 54.3 per 100,000 per year compared to combined ASR of cervical cancer incidence of 34.5 per 100,000 per year. Cervical cancer was the second most common female cancer diagnosed in the country. According to the authors, while the incidence of cervical cancer remained relatively stable, there was a significant increase in the incidence of breast cancer compared to historical records (13.7 per 100,000 women per year for 1960–1969, 24.7 per 100,000 women per year between 1998 and 1999 as shown in Fig. 5 [39, 45]). These data therefore suggested an approximate increase by 80.3% over the four decades and an increase of approximately 20.1% per decade. The recent ASR of 54.3% reported [39] equates to approximately a 120% increase between the years 2000 and 2010. The figure was higher than the GLOBOCAN estimate for 2008 (38.7 per 100,000), although the extent of generalizability might be questioned [39]. Interestingly, the current age-standardized rate, as published by GLOBOCAN in 2012, was 50.5 per 100,000 women per year, placing Nigeria second only to Mauritius (64.2 per 100,000) in Africa.

The most worrisome aspect of this development was the fact that even though Nigeria was ranked second in terms of breast cancer incidence, its age-standardized mortality ratio was the highest in Africa (25.9 per 100,000, compared to 18.8 per 100,000 for Mauritius) (see Fig. 6). Among other factors, the growing cases of breast cancer in Nigeria seemed to have been worsened by the steeply increasing size of the population at risk from approximately 24.5 million in 1990 to approximately 40 million in 2010 and was projected to rise to over 50 million by 2020 [36]. Breast cancer in Nigeria shares similar assumptions that characterise the sub-Saharan African region, i.e. breast cancer cases with late presentation, younger patients and aggressive tumours [34, 44], though these characteristics were still subject to debate. There is an urgent need, therefore, to identify precisely the prevalent risk factors, biological, social and environmental circumstances driving the increasing incidence of breast cancer in Nigeria.

### Implications for regional development

Breast cancer burden will certainly aggravate Africa’s health, socioeconomic and other developmental problems unless action is taken to reduce it. While the continent is still grappling with the burden of infectious diseases, child and maternal deaths (which constitute the top targets for the region in the global effort towards achieving millennium development goals), chronic non-communicable diseases such as cancer are now becoming an increasing threat. The rising burden of non-communicable diseases, including breast cancer, if not tackled in time will overwhelm the coping capacities of African countries which are currently struggling with infectious diseases [46–48].

Cancer had been said to have a greater economic impact than all other diseases with lung cancer, colorectal cancer and breast cancer on the lead [49]. This is bad news for poorly funded health sectors of the African continent [50]. The rising incidence of breast cancer particularly in sub-Saharan Africa deserves urgent attention. It should be noted that breast cancer had been projected to exceed cervical cancer incidence rate in the entire region by 2030 (162,419 new cases compared to 160,163 new cases of cervical cancer) [12]. Experience from within and outside the continent [51–54] strongly suggested that the cost of treating breast cancer is much more than an average household and society in Africa can afford. Although there had not been many studies on the socioeconomic impact of the disease in sub-Saharan African, a 2009 study in Ibadan, Nigeria reported about 85% of those diagnosed with breast cancer were income earners, and of this 70% had an annual income of less than 12,500.00 Nigerian Naira (~ 100USD) [55]. An expert opinion in 2013 reported by one of Nigeria’s leading news dailies showed that a running bill of 150,000–350,000 Nigerian naira (~ $962–$2244, $1 = N156) accumulates for patients every 3 weeks [56]. This was substantially higher than the average income of a Nigerian middle class earner (NGN75, 000–100,000 ($480–645) as well as the national minimum monthly wage of N18, 000($115.4) [57]. The treatment cost in reference was based in Nigerian standard of clinical care. In the USA, individual lifetime lost earnings of $1.10 million had been reported [51] while the estimates of lifetime per-patient direct medical care costs of breast cancer ranged from $US20 000 to $US100000 or more [54, 58]. In Australia, annual household income dropped by $12,000 following breast cancer diagnosis [53]. It is therefore beyond doubt that breast cancer would certainly increase the problem of poverty by depleting household and individual resources in sub-Saharan Africa—a situation that will be worsened by poor or non-existing health insurance for citizens.

Furthermore, the accumulating evidence that breast cancer tends to affect women of higher educational, economical and professional status [59–61] implies that delays in tackling breast cancer could take its toll on the region’s female workforce, especially those holding important societal positions thereby widening the gender gap in employment. It would also constitute a big distraction to their spouses and other family members who might be serving in various strata of the labour force. This would have serious economic consequences. Judging by the total lost productivity cost in advanced countries [51], the economic consequence will take its toll on the already struggling gross domestic product of many African countries. Breast cancer could become another big obstacle to female education in the African continent, as many young females might be taken out of school to either attend to their ailing mothers or take up some of the roles that their mothers might no longer be able to perform due to illness or death [53, 62]. These socioeconomic consequences will be compounded because the sub-Saharan Africa breast cancer cases profile based on available data showed that affected women tended to be younger in age [33, 34]. The economic implication of the disease had been shown to be worse in younger than in old women [52]. The consequence on maternal health will further aggravate the problem of child and infant mortality [63] which is already a big problem in the region.

The high mortality associated with breast cancer in Africa provides clear evidence that the region does not yet have appropriate treatment strategies in place. There was also clear evidence that Africa cannot cope if it allows its current incidence rates to reach the figures of the developed countries of Europe and America. In light of the above, a key line of action for African countries should be to prevent the disease before it happens, through proper identification and exposure reduction to established risk factors; as well as early diagnosis and timely treatment [64].

**Fig. 7:**
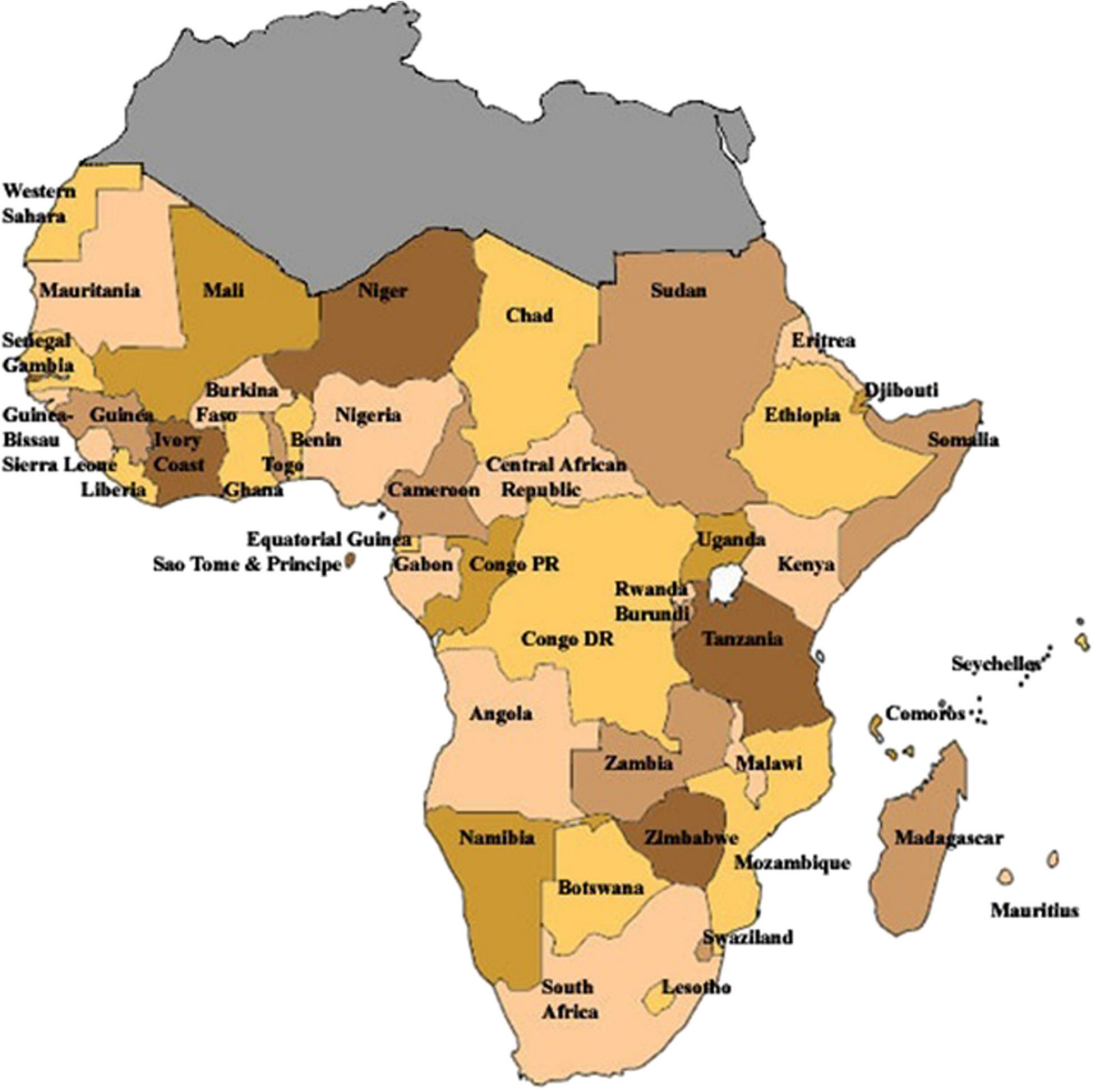
Map of Africa showing sub-Saharan Africa (countries below the grey area). (Source: https://www.librarything.com/topic/183039)

The key question is ‘how can these happen when there are few or no indigenous studies to authenticate the key risk factors involved and the level of risk they pose?’ It is therefore strongly recommended that while efforts for improved diagnosis and treatment continue, research studies should be initiated to identify accurately the risk factors and conditions playing a role in the rising incidence of breast cancer in sub-Saharan Africa (Fig. 7). This will inform the prioritization of interventions in the context of scarce resources.

A possible limitation to this review relates to the general problem of data availability and challenges faced by studies from developing countries. Data availability and good record keeping is a big problem in many African countries. Furthermore, conducting quality research in many African countries is challenging due to poor funding and policy priorities. On the other hand, there seems to be a general consensus among experts that the problem of data unavailability tends to result more in underestimation than overestimation of the problem. This therefore could further strengthen the concerns of this paper. The study strengths further lie in its wide inclusion and elaborate review of key and relevant literature currently available in sub-Saharan Africa.

## Conclusion

This review demonstrates that investment in research to guide interventions for the control of breast cancer in sub-Saharan Africa will have a far-reaching effect. It will help in preserving the gains already made in reduction of child and maternal mortality while sustaining the increasing access to girl/female education in the continent. The avoided social and economic cost will certainly have an unquantifiable benefit to the national development of African countries.

Moreover, the control of breast cancer will provide a great opportunity for further reduction of the existing problem of cervical cancer [64] in sub-Saharan Africa as well as the control of other cancers and non-communicable diseases (e.g. cardiovascular diseases) known to share similar risk factors with breast cancer.
